# Supplementary material for: In Vitro Interactions of Amphiphilic Phosphorous Dendrons with Liposomes and Exosomes—Implications for Blood Viscosity Changes
Source: Pharmaceutics. 2022 Jul 30;14(8):1596. doi: 10.3390/pharmaceutics14081596 (PMC9414926; doi:10.3390/pharmaceutics14081596)
Supplement: Supplementary file 1 [file pharmaceutics-14-01596-s001.zip › pharmaceutics-1822558-supplementary.pdf]

Table S1. Three-way ANOVA results on viscosities of amphiphilic phosphorous dendrons of the first (D1) and second (D2) generation: matching by factors: concentration & dendron generation

Grouping factor	Source of Variation	% of total variation	SS	DF	MS	F (DFn, DFd)	P value
**Shear rate 1 s^{-1}**	Concentration	10.08	127.1	2	63.54	F (2, 20) = 14.08	0.0002
	Dendron generation	0.2578	3.251	1	3.251	F (0.6111, 6.111) = 1.382	0.2496
	Sex	51.05	643.8	1	643.8	F (1, 10) = 20.37	0.0011
	Concentration x Dendron	0.4217	5.318	2	2.659	F (1.456, 14.56) = 1.337	0.283
	Concentration x Sex	0.04126	0.5203	2	0.2601	F (2, 20) = 0.05764	0.9441
	Dendron x Sex	0.5776	7.283	1	7.283	F (1, 10) = 3.095	0.109
	Concentration x Dendron x Sex	0.3352	4.227	2	2.113	F (2, 20) = 1.063	0.3643
	Subject	25.06	316	10	31.6		
	Subject x Concentration	7.158	90.26	20	4.513		
	Subject x Dendron	1.866	23.53	10	2.353		
	Residual		39.78	20	1.1		
**Shear rate 10 s^{-1}**	Concentration	10.56	26.2	2	13.1	F (2, 20) = 14.65	0.0001
	Dendron generation	0.3672	0.9112	1	0.9112	F (0.5152, 5.152) = 1.893	0.1968
	Sex	48.71	120.9	1	120.9	F (1, 10) = 16.54	0.0023
	Concentration x Dendron	0.06749	0.1675	2	0.08375	F (1.672, 16.72) = 0.8242	0.4359
	Concentration x Sex	0.1837	0.4558	2	0.1	F (2, 20) = 0.2550	0.7774
	Dendron x Sex	0.6649	1.65	1	1.65	F (1, 10) = 3.428	0.0938
	Concentration x Dendron x Sex	0.03637	0.09028	2	0.04514	F (2, 20) = 0.4442	0.6475
	Subject	29.45	73.09	10	7.309		
	Subject x Concentration	7.203	17.88	20	0.8938		
	Subject x Dendron	1.939	4.814	10	0.4814		
	Residual		2.032	20	0.1016		
**Shear rate 100 s^{-1}**	Concentration	9.907	6.826	2	3.413	F (2, 20) = 12.30	0.0003
	Dendron generation	0.3066	0.2112	1	0.2112	F (0.5122, 5.122) = 1.875	0.198
	Sex	37.1	25.56	1	25.56	F (1, 10) = 8.906	0.0137
	Concentration x Dendron	0.1318	0.09083	2	0.1	F (1.649, 16.49) = 1.441	0.262
	Concentration x Sex	0.1101	0.07583	2	0.03792	F (2, 20) = 0.1367	0.8731
	Dendron x Sex	0.1695	0.1168	1	0.1168	F (1, 10) = 1.036	0.3326
	Concentration x Dendron x Sex	0.0125	0.008611	2	0.004306	F (2, 20) = 0.1366	0.8732
	Subject	41.66	28.7	10	2.87		
	Subject x Concentration	8.053	5.548	20	0.2774		
	Subject x Dendron	1.636	1.127	10	0.1127		
	Residual		0.6306	20	0.03153		

Abbreviations: SS, sum of squares; DF, degree of freedom; MS, mean squares; F, F statistics; P, probability

Table S2. Three-way ANOVA results on viscosities of amphiphilic phosphorous dendrons of the first (D1) and second (D2) generation: matching by factors: concentration & dendron generation

	Shear rate 1 s⁻¹					Shear rate 10 s⁻¹					Shear rate 100 s⁻¹				
Tukey's multiple comparisons test	Mean 1	Mean 2	Mean Diff	95% CI of diff.	Adjusted P Value	Mean 1	Mean 2	Mean Diff	95% CI of diff.	Adjusted P Value	Mean 1	Mean 2	Mean Diff	95% CI of diff.	Adjusted P Value
c0:D1 Female vs. c0:D1 Male	12.43	17.63	-5.2	-10.03 to -0.3734	0.0327	6.267	8.4	-2.133	-3.796 to -0.4705	0.0142	3.933	4.95	-1.017	-2.155 to 0.1221	0.0835
c0:D1 Female vs. c0:D2 Female	12.43	12.05	0.3833	-0.8668 to 1.633	0.861	6.267	6.3	-0.033	-0.4900 to 0.4234	>0,9999	3.933	3.9	0.033	-0.1850 to 0.2517	0.998
c0:D1 Female vs. c0:D2 Male	12.43	18.72	-6.283	-13.05 to 0.4795	0.0741	6.267	8.9	-2.633	-4.366 to -0.9005	0.0033	3.933	5.117	-1.183	-2.321 to -0.04538	0.0412
c0:D1 Female vs. c1:D1 Female	12.43	14.03	-1.6	-5.308 to 2.108	0.5854	6.267	7.02	-0.75	-2.170 to 0.6698	0.392	3.933	4.267	-0.333	-1.109 to 0.4425	0.5896
c0:D1 Female vs. c1:D1 Male	12.43	19.83	-7.4	-12.36 to -2.442	0.0036	6.267	9.45	-3.183	-5.768 to -0.5986	0.0147	3.933	5.4	-1.467	-3.033 to 0.09923	0.0715
c0:D1 Female vs. c1:D2 Female	12.43	13.83	-1.4	-6.174 to 3.374	0.8851	6.267	6.83	-0.567	-2.498 to 1.365	0.8849	3.933	4.267	-0.333	-1.457 to 0.7907	0.8793
c0:D1 Female vs. c1:D2 Male	12.43	19.63	-7.2	-12.96 to -1.441	0.0121	6.267	9.82	-3.55	-5.367 to -1.733	0.0004	3.933	5.5	-1.567	-2.772 to -0.3610	0.0097
c0:D1 Female vs. c2:D1 Female	12.43	15.38	-2.95	-7.452 to 1.552	0.2211	6.267	7.63	-1.367	-3.270 to 0.5368	0.1663	3.933	4.533	-0.6	-1.536 to 0.3359	0.2359
c0:D1 Female vs. c2:D1 Male	12.43	20.42	-7.983	-12.79 to -3.174	0.0018	6.267	9.93	-3.667	-6.576 to -0.7572	0.0135	3.933	5.717	-1.783	-3.515 to -0.05155	0.0425
c0:D1 Female vs. c2:D2 Female	12.43	15.33	-2.9	-7.399 to 1.599	0.2321	6.267	7.55	-1.283	-4.041 to 1.474	0.5121	3.933	4.65	-0.717	-2.373 to 0.9394	0.5827
c0:D1 Female vs. c2:D2 Male	12.43	22.72	-10.28	-15.70 to -4.871	0.0005	6.267	10.7	-4.383	-7.454 to -1.313	0.0065	3.933	6.017	-2.083	-3.849 to -0.3178	0.0192
c0:D1 Male vs. c0:D2 Female	17.63	12.05	5.583	0.3399 to 10.83	0.0354	8.4	6.3	2.1	0.3852 to 3.815	0.0183	4.95	3.9	1.05	0.07515 to 2.025	0.0344
c0:D1 Male vs. c0:D2 Male	17.63	18.72	-1.083	-6.771 to 4.604	0.9899	8.4	8.9	-0.5	-2.132 to 1.132	0.8615	4.95	5.117	-0.167	-0.9071 to 0.5738	0.9707
c0:D1 Male vs. c1:D1 Female	17.63	14.03	3.6	-2.729 to 9.929	0.4346	8.4	7.02	1.383	-1.501 to 4.268	0.5221	4.95	4.267	0.683	-1.134 to 2.501	0.7583
c0:D1 Male vs. c1:D1 Male	17.63	19.83	-2.2	-8.506 to 4.106	0.7739	8.4	9.45	-1.05	-3.209 to 1.109	0.4695	4.95	5.4	-0.45	-1.652 to 0.7515	0.716
c0:D1 Male vs. c1:D2 Female	17.63	13.83	3.8	-3.682 to 11.28	0.5315	8.4	6.83	1.567	-1.916 to 5.049	0.5744	4.95	4.267	0.683	-1.452 to 2.819	0.8623
c0:D1 Male vs. c1:D2 Male	17.63	19.63	-2	-6.322 to 2.322	0.5176	8.4	9.82	-1.417	-2.750 to -0.08350	0.0392	4.95	5.5	-0.55	-1.174 to 0.07357	0.0811
c0:D1 Male vs. c2:D1 Female	17.63	15.38	2.25	-5.254 to 9.754	0.9372	8.4	7.63	0.7667	-2.537 to 4.071	0.9734	4.95	4.533	0.417	-1.568 to 2.402	0.9869
c0:D1 Male vs. c2:D1 Male	17.63	20.42	-2.783	-8.339 to 2.772	0.4411	8.4	9.93	-1.533	-4.156 to 1.089	0.3047	4.95	5.717	-0.767	-2.198 to 0.6651	0.3797
c0:D1 Male vs. c2:D2 Female	17.63	15.33	2.3	-5.797 to 10.40	0.9494	8.4	7.55	0.85	-3.454 to 5.154	0.9895	4.95	4.65	0.3	-2.419 to 3.019	>0,9999
c0:D1 Male vs. c2:D2 Male	17.63	22.72	-5.083	-12.81 to 2.640	0.2182	8.4	10.7	-2.25	-5.139 to 0.6387	0.1267	4.95	6.017	-1.067	-2.554 to 0.4202	0.1668
c0:D2 Female vs. c0:D2 Male	12.05	18.72	-6.667	-13.60 to 0.2623	0.062	6.3	8.9	-2.6	-4.375 to -0.8252	0.0044	3.9	5.117	-1.217	-2.197 to -0.2367	0.0151
c0:D2 Female vs. c1:D1 Female	12.05	14.03	-1.983	-4.834 to 0.8669	0.1839	6.3	7.02	-0.717	-2.255 to 0.8219	0.5112	3.9	4.267	-0.367	-1.346 to 0.6128	0.7165
c0:D2 Female vs. c1:D1 Male	12.05	19.83	-7.783	-13.13 to -2.440	0.0046	6.3	9.45	-3.15	-5.747 to -0.5527	0.016	3.9	5.4	-1.5	-3.018 to 0.01767	0.0533
c0:D2 Female vs. c1:D2 Female	12.05	13.83	-1.783	-5.516 to 1.950	0.4866	6.3	6.83	-0.533	-2.586 to 1.519	0.9353	3.9	4.267	-0.367	-1.654 to 0.9204	0.8991
c0:D2 Female vs. c1:D2 Male	12.05	19.63	-7.583	-13.60 to -1.563	0.0114	6.3	9.82	-3.517	-5.370 to -1.663	0.0005	3.9	5.5	-1.6	-2.688 to -0.5116	0.0037

Tukey's multiple comparisons test	Shear rate 1 s^{-1}					Shear rate 10 s^{-1}					Shear rate 100 s^{-1}				
	Mean 1	Mean 2	Mean Diff	95% CI of diff.	Adjusted P Value	Mean 1	Mean 2	Mean Diff	95% CI of diff.	Adjusted P Value	Mean 1	Mean 2	Mean Diff	95% CI of diff.	Adjusted P Value
c0:D2 Female vs. c2:D1 Female	12.05	15.38	-3.333	-6.967 to 0.3006	0.07	6.3	7.63	-1.333	-3.292 to 0.6257	0.197	3.9	4.533	-0.633	-1.773 to 0.5064	0.3472
c0:D2 Female vs. c2:D1 Male	12.05	20.42	-8.367	-13.60 to -3.136	0.0027	6.3	9.93	-3.633	-6.550 to -0.7163	0.0143	3.9	5.717	-1.817	-3.516 to -0.1169	0.035
c0:D2 Female vs. c2:D2 Female	12.05	15.33	-3.283	-6.978 to 0.4110	0.0788	6.3	7.55	-1.25	-4.114 to 1.614	0.5744	3.9	4.65	-0.75	-2.582 to 1.082	0.6357
c0:D2 Female vs. c2:D2 Male	12.05	22.72	-10.67	-16.39 to -4.948	0.0006	6.3	10.7	-4.35	-7.426 to -1.274	0.0068	3.9	6.017	-2.117	-3.853 to -0.3805	0.0166
c0:D2 Male vs. c1:D1 Female	18.72	14.03	4.683	-2.800 to 12.17	0.3904	8.9	7.02	1.883	-0.9558 to 4.722	0.2611	5.117	4.267	0.85	-0.9585 to 2.659	0.5623
c0:D2 Male vs. c1:D1 Male	18.72	19.83	-1.117	-10.98 to 8.748	0.9998	8.9	9.45	-0.55	-4.072 to 2.972	0.9977	5.117	5.4	-0.283	-2.104 to 1.537	0.9977
c0:D2 Male vs. c1:D2 Female	18.72	13.83	4.883	-3.335 to 13.10	0.4472	8.9	6.83	2.067	-1.357 to 5.490	0.3286	5.117	4.267	0.85	-1.276 to 2.976	0.7052
c0:D2 Male vs. c1:D2 Male	18.72	19.63	-0.9167	-5.772 to 3.938	0.9905	8.9	9.82	-0.917	-2.757 to 0.9235	0.4466	5.117	5.5	-0.383	-1.342 to 0.5754	0.6576
c0:D2 Male vs. c2:D1 Female	18.72	15.38	3.333	-4.900 to 11.57	0.8442	8.9	7.63	1.267	-1.981 to 4.515	0.7669	5.117	4.533	0.583	-1.392 to 2.559	0.9139
c0:D2 Male vs. c2:D1 Male	18.72	20.42	-1.7	-9.967 to 6.567	0.9832	8.9	9.93	-1.033	-4.922 to 2.855	0.9275	5.117	5.717	-0.6	-2.650 to 1.450	0.886
c0:D2 Male vs. c2:D2 Female	18.72	15.33	3.383	-5.274 to 12.04	0.8635	8.9	7.55	1.35	-2.891 to 5.591	0.8807	5.117	4.65	0.467	-2.243 to 3.177	0.9965
c0:D2 Male vs. c2:D2 Male	18.72	22.72	-4	-14.26 to 6.263	0.6809	8.9	10.7	-1.75	-5.548 to 2.048	0.5216	5.117	6.017	-0.9	-2.883 to 1.083	0.5365
c1:D1 Female vs. c1:D1 Male	14.03	19.83	-5.8	-12.17 to 0.5719	0.0802	7.017	9.45	-2.433	-5.577 to 0.7106	0.1809	4.267	5.4	-1.133	-3.068 to 0.8010	0.4604
c1:D1 Female vs. c1:D2 Female	14.03	13.83	0.2	-2.699 to 3.099	>0,9999	7.017	6.83	0.1833	-0.9701 to 1.337	0.9973	4.267	4.267	0	-0.6127 to 0.6127	>0,9999
c1:D1 Female vs. c1:D2 Male	14.03	19.63	-5.6	-12.39 to 1.195	0.1356	7.017	9.82	-2.8	-5.642 to 0.04224	0.0539	4.267	5.5	-1.233	-3.027 to 0.5602	0.243
c1:D1 Female vs. c2:D1 Female	14.03	15.38	-1.35	-2.959 to 0.2587	0.0974	7.017	7.63	-0.617	-1.206 to -0.02750	0.0416	4.267	4.533	-0.267	-0.4393 to -0.09405	0.0078
c1:D1 Female vs. c2:D1 Male	14.03	20.42	-6.383	-12.71 to -0.05884	0.0477	7.017	9.93	-2.917	-6.256 to 0.4227	0.1039	4.267	5.717	-1.45	-3.481 to 0.5810	0.251
c1:D1 Female vs. c2:D2 Female	14.03	15.33	-1.3	-4.548 to 1.948	0.6566	7.017	7.55	-0.533	-2.305 to 1.239	0.8715	4.267	4.65	-0.383	-1.405 to 0.6386	0.7147
c1:D1 Female vs. c2:D2 Male	14.03	22.72	-8.683	-15.27 to -2.093	0.009	7.017	10.7	-3.633	-7.079 to -0.1877	0.0365	4.267	6.017	-1.75	-3.802 to 0.3022	0.1169
c1:D1 Male vs. c1:D2 Female	19.83	13.83	6	-1.488 to 13.49	0.1368	9.45	6.83	2.617	-0.9524 to 6.186	0.2184	5.4	4.267	1.133	-1.039 to 3.306	0.5791
c1:D1 Male vs. c1:D2 Male	19.83	19.63	0.2	-7.217 to 7.617	>0,9999	9.45	9.82	-0.367	-2.384 to 1.651	0.9927	5.4	5.5	-0.1	-1.092 to 0.8916	>0,9999
c1:D1 Male vs. c2:D1 Female	19.83	15.38	4.45	-3.060 to 11.96	0.3823	9.45	7.63	1.817	-1.618 to 5.252	0.5755	5.4	4.533	0.867	-1.189 to 2.923	0.8033
c1:D1 Male vs. c2:D1 Male	19.83	20.42	-0.5833	-2.928 to 1.761	0.9484	9.45	9.93	-0.483	-1.333 to 0.3667	0.3276	5.4	5.717	-0.317	-0.7046 to 0.07124	0.1075
c1:D1 Male vs. c2:D2 Female	19.83	15.33	4.5	-3.590 to 12.59	0.4385	9.45	7.55	1.9	-2.348 to 6.148	0.7179	5.4	4.65	0.75	-1.920 to 3.420	0.9683
c1:D1 Male vs. c2:D2 Male	19.83	22.72	-2.883	-5.118 to -0.6483	0.0173	9.45	10.7	-1.2	-2.583 to 0.1831	0.0862	5.4	6.017	-0.617	-1.088 to -0.1455	0.0162
c1:D2 Female vs. c1:D2 Male	13.83	19.63	-5.8	-13.52 to 1.922	0.1952	6.833	9.82	-2.983	-6.395 to 0.4281	0.0917	4.267	5.5	-1.233	-3.330 to 0.8637	0.371
c1:D2 Female vs. c2:D1 Female	13.83	15.38	-1.55	-3.794 to 0.6943	0.1883	6.833	7.63	-0.8	-2.103 to 0.5032	0.267	4.267	4.533	-0.267	-0.8745 to 0.3412	0.5694
c1:D2 Female vs. c2:D1 Male	13.83	20.42	-6.583	-14.07 to 0.8992	0.0898	6.833	9.93	-3.1	-6.808 to 0.6082	0.1274	4.267	5.717	-1.45	-3.692 to 0.7923	0.3457
c1:D2 Female vs. c2:D2 Female	13.83	15.33	-1.5	-4.068 to 1.068	0.3057	6.833	7.55	-0.717	-1.556 to 0.1228	0.0915	4.267	4.65	-0.383	-0.9725 to 0.2058	0.2258

Tukey's multiple comparisons test	Shear rate 1 s^{-1}					Shear rate 10 s^{-1}					Shear rate 100 s^{-1}				
	**Mean 1**	**Mean 2**	**Mean Diff**	**95% CI of diff.**	**Adjusted P Value**	**Mean 1**	**Mean 2**	**Mean Diff**	**95% CI of diff.**	**Adjusted P Value**	**Mean 1**	**Mean 2**	**Mean Diff**	**95% CI of diff.**	**Adjusted P Value**
c1:D2 Female vs. c2:D2 Male	13.83	22.72	-8.883	-16.48 to -1.289	0.0206	6.833	10.7	-3.817	-7.605 to -0.02854	0.0479	4.267	6.017	-1.75	-4.008 to 0.5083	0.1781
c1:D2 Male vs. c2:D1 Female	19.63	15.38	4.25	-3.491 to 11.99	0.5164	9.817	7.63	2.183	-1.056 to 5.423	0.2505	5.5	4.533	0.967	-0.9854 to 2.919	0.5631
c1:D2 Male vs. c2:D1 Male	19.63	20.42	-0.7833	-6.513 to 4.946	0.9992	9.817	9.93	-0.117	-2.360 to 2.126	>0,9999	5.5	5.717	-0.217	-1.423 to 0.9898	0.9933
c1:D2 Male vs. c2:D2 Female	19.63	15.33	4.3	-3.948 to 12.55	0.5646	9.817	7.55	2.267	-1.952 to 6.486	0.4379	5.5	4.65	0.85	-1.820 to 3.520	0.891
c1:D2 Male vs. c2:D2 Male	19.63	22.72	-3.083	-11.62 to 5.457	0.7468	9.817	10.7	-0.833	-2.886 to 1.219	0.6434	5.5	6.017	-0.517	-1.615 to 0.5812	0.5012
c2:D1 Female vs. c2:D1 Male	15.38	20.42	-5.033	-12.54 to 2.471	0.253	7.633	9.93	-2.3	-5.890 to 1.290	0.3615	4.533	5.717	-1.183	-3.321 to 0.9545	0.5311
c2:D1 Female vs. c2:D2 Female	15.38	15.33	0.05	-2.109 to 2.209	>0,9999	7.633	7.55	0.0833	-1.600 to 1.766	>0,9999	4.533	4.65	-0.117	-1.037 to 0.8040	0.9996
c2:D1 Female vs. c2:D2 Male	15.38	22.72	-7.333	-14.95 to 0.2809	0.0609	7.633	10.7	-3.017	-6.694 to 0.6606	0.1403	4.533	6.017	-1.483	-3.640 to 0.6729	0.2856
c2:D1 Male vs. c2:D2 Female	20.42	15.33	5.083	-3.017 to 13.18	0.304	9.933	7.55	2.383	-1.943 to 6.709	0.5119	5.717	4.65	1.067	-1.636 to 3.770	0.8327
c2:D1 Male vs. c2:D2 Male	20.42	22.72	-2.3	-5.648 to 1.048	0.1914	9.933	10.7	-0.717	-1.781 to 0.3481	0.2039	5.717	6.017	-0.3	-0.8828 to 0.2828	0.4158
c2:D2 Female vs. c2:D2 Male	15.33	22.72	-7.383	-15.54 to 0.7694	0.082	7.55	10.7	-3.1	-7.476 to 1.276	0.2475	4.65	6.017	-1.367	-4.078 to 1.345	0.6051
